# Introducing the GEV Activation Function for Highly Unbalanced Data to Develop COVID-19 Diagnostic Models

**DOI:** 10.1109/JBHI.2020.3012383

**Published:** 2020-07-28

**Authors:** Joshua Bridge, Yanda Meng, Yitian Zhao, Yong Du, Mingfeng Zhao, Renrong Sun, Yalin Zheng

**Affiliations:** 1 Institute of Life Course and Medical SciencesUniversity of Liverpool 4591 Liverpool L7 8TX U.K.; 2 Cixi Institute of Biomedical EngineeringNingbo Institute of Materials Technology and Engineering, Chinese Academy of Sciences 74748 Ningbo 315201 China; 3 Department of Nuclear MedicineThe Royal Marsden NHS Foundation Trust 4970 Sutton SM2 5PT U.K.; 4 Department of HematologyTianjin First Central Hospital, the First Central Clinical College of Tianjin Medical University 66558 Tianjin 300192 China; 5 Department of RadiologyHubei Provincial Hospital of Integrated Chinese and Western Medicine, Hubei University of Chinese Medicine 240515 Wuhan 430000 China

**Keywords:** Artificial intelligence, computer-aided detection and diagnosis, covid-19, extreme value theory, lung, x-ray and computed tomography

## Abstract

Fast and accurate diagnosis is essential for the efficient and effective control of the COVID-19 pandemic that is currently disrupting the whole world. Despite the prevalence of the COVID-19 outbreak, relatively few diagnostic images are openly available to develop automatic diagnosis algorithms. Traditional deep learning methods often struggle when data is highly unbalanced with many cases in one class and only a few cases in another; new methods must be developed to overcome this challenge. We propose a novel activation function based on the generalized extreme value (GEV) distribution from extreme value theory, which improves performance over the traditional sigmoid activation function when one class significantly outweighs the other. We demonstrate the proposed activation function on a publicly available dataset and externally validate on a dataset consisting of 1,909 healthy chest X-rays and 84 COVID-19 X-rays. The proposed method achieves an improved area under the receiver operating characteristic (DeLong's p-value < 0.05) compared to the sigmoid activation. Our method is also demonstrated on a dataset of healthy and pneumonia vs. COVID-19 X-rays and a set of computerized tomography images, achieving improved sensitivity. The proposed GEV activation function significantly improves upon the previously used sigmoid activation for binary classification. This new paradigm is expected to play a significant role in the fight against COVID-19 and other diseases, with relatively few training cases available.

## I. Introduction

Covid-19 is an acute respiratory syndrome with over 6.4 million cases as of 4 June 2020, with the number of cases rapidly increasing, and over 382,000 deaths reported worldwide [1]. Fast and accurate diagnosis is essential for the efficient and effective control of this pandemic. Reverse-transcription polymerase chain reaction (RT-PCR) is the current standard test for the COVID-19. RT-PCR has been found to have a variable sensitivity with a low value of 71% when compared to computerized tomography (CT) with a sensitivity of 98% (p < 0.01) [2], leading to a significant number of false negatives. Currently, there are no other laboratory tests able to provide timely results with such high sensitivity.

The World Health Organization has outlined several scenarios in which laboratory testing may not be sufficient and chest imaging is needed [3]. Medical imaging is quickly becoming vital in the diagnosis of COVID-19 as imaging is faster, cheaper, and more readily available compared to RT-PCR. CT based COVID-19 diagnosis is reported to have a higher sensitivity (80–90%) when compared to laboratory tests at the expense of specificity (60–70%) [4]. Chest X-ray is a cheaper alternative to CT with a faster image acquisition time; X-Ray equipment is also easier to sanitize compared to CT machines. Chest X-ray is of most use in low income, low resource settings, such as developing countries or community screening programs. A study into the radiological features of COVID-19 [5], reported that the most significant feature is ground-glass opacification (GGO), sometimes accompanied by consolidation in more severe forms [6]. In some cases, these features were observable on CT but not on X-ray imaging. The authors concluded that CT might be more sensitive to these features than X-ray.

Given that COVID-19 is a relatively new condition, there are currently no experienced COVID-19 radiologists compared to other established conditions, and there is little time for training. The disease affects a large number of patients within a short period of time, and as a result, experts are overladen with the number of cases requiring imaging and diagnosis; this is likely to become worse as countries look to increase testing. With such a rapid rate of infection, a new, fast, and accurate method of COVID-19 diagnosis is required. Although some countries, such as China, have been successful in controlling the spread of the virus, it is feared that, as restrictions are relaxed, the virus will become established in populations; therefore, a robust and effective strategy to diagnose COVID-19 must be found. Moreover, imaging may play an essential role in assessing the disease response to treatment, further facilitating the urgently needed development of effective treatments.

The automated diagnosis of COVID-19 is an active research area as surveyed by Shi *et al.* [7] and Wynants *et al.* [8]. Despite the high prevalence of the virus, relatively few COVID-19 images are available to build a deep learning automatic diagnosis model, compared to the availability of healthy or non-COVID-19 images. If all available images are used, then COVID-19 cases will be under-represented, potentially resulting in many false negatives. This echoes the common issue of unbalanced data in medical image analysis. Previous methods to combat this problem primarily rely on resampling the under-represented data or reweighting the loss function [9]; this can lead to overfitting or can significantly increase training time. Here, we propose a novel activation function based on the generalized extreme value (GEV) distribution in extreme value theory [10] to address this fundamental problem. The proposed GEV activation function is better able to model the tail of unbalanced data, where one class has relatively few cases when compared to the other.

We first show the benefit of our proposed activation function over sigmoid in a balanced dataset of X-ray images. We report performance at different level of imbalance and show improved results over using sigmoid and using oversampling strategy with the sigmoid activation. We then demonstrate the GEV activation function by developing three prediction models to diagnose COVID-19. The first model aims to diagnose COVID-19 from a set consisting of healthy vs. COVID-19 X-ray images; these images may be observed in a screening program. The second model diagnoses COVID-19 from a set of COVID-19 vs. healthy and pneumonia X-ray images. The final model classifies CT images as COVID-19 or non-COVID-19, which may have other diseases present.

The GEV activation function can achieve excellent performance with relatively few positive training images. By reducing the number of COVID-19 images needed for training, we can use more images in the testing to better assess the generalizability of the model. The models developed here are to demonstrate the strength of the proposed activation function, and we believe that our proposed GEV based model will form the basis for further development towards real-world clinic use after proper clinical evaluation.

### A. Contributions

Our main contributions are in both methodology and applications, for deep learning and medical image analysis, we propose an activation function based on the GEV distribution, which aims to model the distribution of unbalanced data better. To the best of our knowledge, this is the first time such an activation function has been utilized within deep learning. We demonstrate the added benefit of the GEV activation by comparing it to the classic sigmoid activation, with improved sensitivity. We show that the proposed activation reduces the number of images required in training. The GEV function can be used with any convolutional neural network (CNN) and alongside other unbalanced data methods, such as class weights. In terms of applications, we have applied the new activation function to the diagnosis of COVID-19 using X-Rays and CT images and made some groundbreaking work for the future development of fast and accurate diagnosis solutions of COVID-19.

## II. Related Work

In this section, we provide a concise and thorough overview of the related work in the diagnosis of COVID-19 using X-ray and CT images, attempts to overcome the unbalanced data problem, and general uses of extreme value theory.

Deep learning-based methods have previously been used to diagnose a variety of diseases from medical images accurately, often outperforming human graders [11]. More recently, deep learning has been applied to medical images to detect COVID-19 [7]. For brevity, we do not consider papers focusing solely on COVID-19 segmentation; readers interested in segmentation may wish to read a recent review of AI in COVID-19 [7].

### A. X-Ray Diagnosis

Wang *et al.* [12] developed a custom CNN with a residual architecture, to classify images as COVID-19, pneumonia, or healthy. The model was trained on publicly available images from two datasets [13], [14]. With only 10 COVID-19 images in the testing dataset, the model attained a sensitivity of 0.80 and a specificity of 0.995. This work suggests that future models need to improve sensitivity to reduce the number of false negatives.

A Bayesian convolutional neural network (BCNN) with drop weights, based on ResNet-50 V2 [15], was proposed by Ghoshal *et al.* [16]. Data consisted of four classes, normal, bacterial pneumonia, non-COVID-19 viral pneumonia, and COVID-19. A total of 14 COVID-19 images were used in the testing dataset. In both CNNs and BCNNs with a range of drop weights, two of the COVID-19 cases were wrongly classified. For COVID-19 diagnosis, their best model attained a sensitivity of 0.857 and a specificity of 0.995. Various saliency maps were used to reduce the black-box nature of deep learning; however, the maps appeared to highlight some wrong areas such as the collarbone, and this was not explained or investigated within the paper. The study concluded that by estimating uncertainty within predictions, model performance can be improved.

Narin *et al.* [17] obtained 50 healthy and 50 COVID-19 patients and used three different pretrained deep learning networks to diagnose COVID-19. Using ten images from each class in a testing set, they obtained perfect performance in using Inception V3 [18] and ResNet-50 [19], with Inception-ResNet V2 [20] misclassifying one of the healthy images as COVID-19. They suggested that transfer learning can be used to build deep learning models for COVID-19 diagnosis. An 18-layer residual CNN pretrained on ImageNet [21] was proposed by Zhang *et al.* [22]. The CNN was followed by fully-connected layers and a sigmoid activation for classification. A separate anomaly detection mechanism was also added to the end of the CNN. Sensitivity and specificity were reported at different thresholds, the model achieves a sensitivity of 0.72 at 0.98 specificity and a sensitivity of 0.966 at 0.707 specificity, in two-fold cross-validation of 100 images from 70 patients.

In summary, these models appear to perform very well; however, increasing the sensitivity of models is a priority due to the risk associated with missing a COVID-19 diagnosis.

### B. CT Diagnosis

There have been more models developed using CT to diagnose COVID-19, many are reviewed in [8] and [7].

Chen *et al.* [23] used a UNet++ [24] based approach for the detection of COVID-19 lesions and then for the diagnosis of COVID-19. The model was trained on expert annotated CT slices to extract COVID-19 areas. They concluded that their algorithm has comparable performance to expert radiologists, with a reported sensitivity of 1.0 on the patient level, with 16 suspected pneumonia and 11 COVID-19 patients. Shan *et al.* [25] used a similar method with a different network to segment and quantify COVID-19, achieving a dice score of 0.916, with 300 COVID-19 patients. The authors proposed that their method could be used to analyze the progression of the disease.

Gozes *et al.* [26] combined commercial software and deep learning to segment and quantify COVID-19, with a reported area under the receiver operating characteristic (AUC) of 0.996. Li *et al.* [27] used a ResNet-50 network for each slice in a CT image, with shared weights. Max pooling then combined the slices to provide a single feature vector, which was then classified. The method achieved an AUC of 0.96, with 285 healthy and 68 COVID-19 patients. A patch-based method with a support vector machine was proposed by Barstugan *et al.* [28]; this achieved a specificity of 1.0 and a sensitivity of 0.93 using a cross-validation of 53 patients.

Shi *et al.* [29] segmented scans to extract infection and lung fields, an infection size aware random forest classifier then classified images according to infection size. The method attained an overall AUC of 0.94, with 1,027 healthy and 1,658 COVID-19 patients using 5-fold cross validation. Jin *et al.* [30] first segmented lesions using a 3D neural network, before classifying those lesions as COVID-19 or not, with a 2D ResNet network. Data from two hospitals were used and this method achieved an AUC of 0.99 on 128 healthy and 154 COVID-19 patients. Jin *et al.* [31] used a segmentation network (Deeplab v1, [32]) and a ResNet152 classification network for the classification of CT slices, GradCAMs [33] were then produced to highlight the diseased area. This network was trained on local and public datasets, achieving an AUC of 0.98 on 1,072 healthy and 183 COVID-19 patients. Xu *et al.* [34] proposed using a deep learning model to segment out the candidate infection regions. Patches of infected regions, with the distances from the edge of the lung, were input to a ResNet-18 network for classification into three groups: COVID-19, Influenza-A, and healthy patients. The model achieves an overall accuracy of 0.867 on 30 COVID-19 patients and 60 other patients.

Wang *et al.* [35] have applied an inception network for the diagnosis of COVID-19 using an in-house dataset. They reported a total accuracy of 89.5% with a specificity of 0.88 and a sensitivity of 0.87 in the internal validation, and a total accuracy of 0.793 with a specificity of 0.83 and a sensitivity of 0.67 in the external validation set. The external validation set consisted of 100 healthy patients, 100 pneumonia patients, and 10 COVID-19 patients. Song *et al.* [36] proposed using ResNet-50, and a feature pyramid network (FPN) combined with an attention module and experimented on their in-house dataset. An AUC of 0.99 and a sensitivity of 0.93 for COVID19 and healthy images were reported, on a set of 24 healthy and 27 COVID-19 patients. In a dataset of 30 pneumonia 27 COVID-19 patients, their method attained an AUC of 0.95 and a sensitivity of 0.96 for COVID and bacteria pneumonia-infected patients.

Tang *et al.* [37] used a random forest (RF) model to assess the severity of COVID-19 based on quantitative features derived from a deep learning model. With three-fold cross-validation on 176 patients the method attained an overall accuracy of 0.875. A weakly supervised method was proposed by Zheng *et al.* [38]. Their method automatically generated segmentation masks. The CT image and mask are fed into a 3D CNN for classification. This method attained an AUC of 0.959.

In summary, similar to the studies using X-ray imaging, most studies use a small number of COVID-19 images from different resources without standardized protocols. It seems that they are just applications of existing AI tools to a new problem, and thus novelty in AI and clinical usefulness are limited. The high data heterogeneity among the studies makes it difficult to compare with. Although all models achieved excellent performance, Wynants *et al.* [8] found that the risk of bias high in all the eight studies that they have reviewed, according to PROBAST [39].

Overall models for COVID-19 diagnosis, using X-ray or CT images, attain excellent performance; however, some models only use as few as 10 COVID-19 images within the testing set, and few models use external validation primarily due to the issue of data availability. Therefore they may not necessarily be generalizable to other cases. A method that is more data-efficient, attaining high performance with fewer training images is needed; this would allow more images of the rare class to be used in the testing data. The aim of our work is not necessarily to beat these previous models but to provide a method which may improve the previous models.

### C. Unbalanced Data

When a dataset has only a few classes containing the majority of the data and many classes that only occur a few times, the data distribution becomes long-tailed. It has been observed that the classes with more samples have a greater effect on the learned features [40]. The most frequent classes become much easier to model, and the tail of the distribution, consisting of rarer classes, is not adequately modeled [41]. This issue exists in both binary and multiclass problems and remains a major challenge in data science.

There are many published methods aimed at addressing the issue of unbalanced data by better modeling the tail of the data distribution. Several papers have proposed reweighting the loss function [42] or oversampling under-represented classes. Other methods include utilizing representation learning [41], learning features by clustering classes into visually similar groups [40], and introducing a loss function to increase the within-subject variation [22].

More recently, Cui *et al.* [9] proposed a new loss function that exploits the information overlap within the data. They argue that although more data will increase the information available, the marginal benefit will decrease with each new sample due to information within the data overlapping. The authors provide a theoretical framework for quantifying the data overlap so that the theoretical effective number of samples. The effective number of samples allows samples from the training set to rebalance the data slightly; however, this does not guarantee that the data will then be sufficiently balanced.

### D. Extreme Value Distributions

Deep learning classification networks often consist of a CNN followed by classification layers comprised of a fully connected (FC) layer and an activation function, usually sigmoid for binary classification and softmax for multiclass classification. When the sigmoid activation function is used, then the classification layers become analogous to a generalized linear model with logistic link function, also called logistic regression. Logistic regression uses the sigmoid function as a link function and assumes that the errors follow the logistic distribution; however, this assumption is not valid in highly skewed or unbalanced data.

In highly unbalanced data, it is vital to model the tail and skewness of the distribution appropriately, to avoid introducing bias. A simulation study by Czado and Santner [43] found that a highly mispecified link function greatly increases bias in both the parameter estimates and the predicted probability. A highly skewed distribution such as the GEV distribution can be used to properly describe the distribution, ensuring that bias within the model is reduced.

In traditional statistics for problems such as finance, weather, or epilepsy [44], [45], link functions based on extreme value distributions [10] have been proposed for when data is highly unbalanced. Extreme value distributions assume that the errors follow a highly skewed distribution, such as those observed when data has some rare classes.

Extreme value theory has previously been used in deep learning to infer labels from intermediate CNN features from a bag of 3D image volumes, in multiple-instance learning [46]. Our work differs significantly by using the generalized extreme value distribution to create a new activation function, which improves classification, in particular sensitivity, when one class has significantly fewer cases.

## III. Methods

**Fig. 1.**
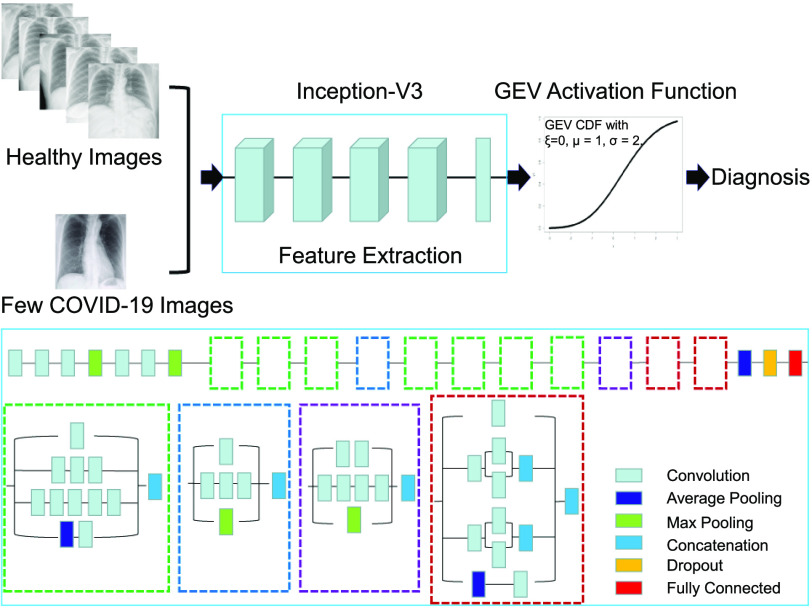
Overview of the general framework. The dataset is highly unbalanced, with one class significantly outweighing the other. A CNN is used to extract features from the images. A fully connected layer then reduces the features to a single value using a linear combination. The GEV activation function converts that value to a probability.

We first describe the general network in which the proposed activation function may be used. We then describe the proposed activation function and its implementation. An overview of the framework is shown in Fig. 1.

### A. CNN

The proposed activation function may be added to any neural network, including pretrained networks such as ResNet [15], [19] or more novel networks such as COVID-NET [12]. Here, we begin with a pretrained CNN, namely Inception V3 [18]. Inception V3 is a variation of the original inception network with improvements in accuracy and a reduction in computational complexity. Inception V3 is commonly used in classification problems, due to its high generalizability. Inception V3 attains high accuracy while maintaining reasonable computational efficiency through its use of batch normalization and heavy regularization. In brief, Inception V3 network stacks 11 inception blocks where each block consists of convolution filters and pooling filters with rectified linear units as activation functions. The input of the model is a two-dimensional image with three channels. At the end of the model, we apply a global averaging pooling layer and add a fully connected layer of size 2048. A dropout rate of 0.6 is used to reduce overfitting. Transfer learning is utilized to reduce training time, with the network pretrained on Imagenet [21]. The model is then fine-tuned on our datasets.

### B. Activation Functions

The proposed GEV activation function is implemented as a network layer with three trainable parameters, $\xi$, $\mu$, $\sigma$. Extreme value distributions are designed explicitly for long-tailed distributions, such as those produced when one class has relatively few cases [10]. Extreme value theory allows us to better model the tail of the distribution than the traditionally used sigmoid activation. The GEV distribution cumulative distribution function (CDF) is given by:

\begin{equation*}
F(x|\xi, \mu, \sigma) = {\begin{cases}\exp (-\exp (\frac{x-\mu }{\sigma })), & \text{if } \xi = 0,\\
\exp (-(1+\xi \left(\frac{x-\mu }{\sigma }\right)^{-1/\xi }), & \text{if } \xi \ne 0 .\\
\end{cases}} \tag{1}
\end{equation*}

This distribution is a generalization of three extreme value distributions. When $\xi = 0$, the GEV becomes the Gumbel distribution, when $\xi >0$, the GEV becomes the Frechet distribution, and when $\xi < 0$, the GEV becomes the Weibull distribution.

To assess the added benefit of the proposed activation function, we compare the proposed activation function with the previously used sigmoid function, given by:

\begin{equation*}
\sigma (x)=\frac{1}{1+\exp (-x)}.
\end{equation*}

**Fig. 2.**
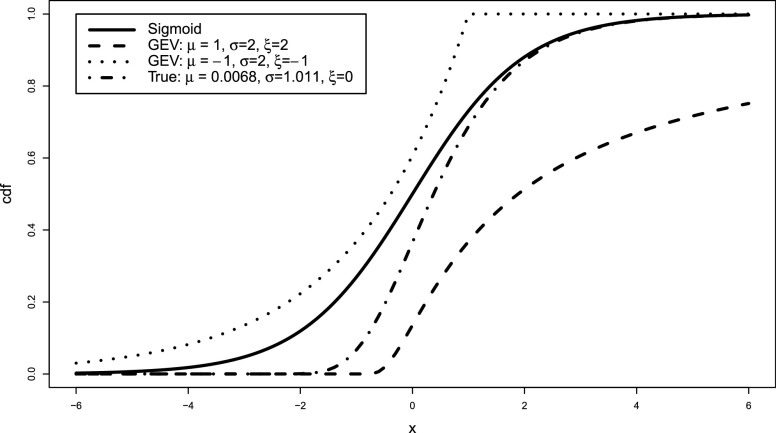
Example curves of the sigmoid curve, and GEV distribution cumulative distribution functions (CDFs). True indicates the actual curve learned from the toy pneumonia dataset at a imbalance ratio of 1:50.

Examples showing the sigmoid curve and three types of GEV CDF are shown in Fig. 2. The three parameters allow the GEV to adapt. Small changes within the parameters can lead to large differences in predictions. The GEV activation function has three trainable parameters that change the shape and scale of the curve. In traditional statistics, the GEV parameters would be estimated using maximum likelihood [47], which requires a full rank design matrix; however, in a deep learning context, these parameters can be learned through gradient descent along with the other model parameters. This only adds an extra three parameters to be trained.

### C. Datasets

#### 1) Balanced Dataset

To evaluate our activation function we first look at a widely used toy dataset related to our main aim. The dataset consists of community acquired pneumonia and normal X-ray images [48]. We begin by balancing the data to 1,583 in each class giving a ratio of 1:1. We split the data into 800 images for training, 200 images for validation, and 583 images for testing, from each class. We then reduce the number of pneumonia images in the training and validation sets, with testing remaining constant. Pneumonia images were removed to give pneumonia:normal ratios of 1:10, 1:25 and 1:50.

#### 2) X-Ray Datasets

COVID-19 positive X-Rays were obtained from the COVID-19 image data collection [13], consisting of images from a variety of sources such as case notes and publications. In the training and validation, we used images from the Italian Society of Radiology [49] only, with images obtained from other sources used for testing/external validation. Healthy X-rays for training and validation were taken from the ChestX-Ray8 [14], with testing/external validation images taken from Kermany *et al.* [48] and the Shenzhen Hospital X-Ray dataset [50].

Any images with distinguishing annotations/artifacts that could not be cropped out or less than 256 pixels in either height or width were removed. After this, we were left with 30 COVID-19 and 40,240 healthy images in the training dataset, 15 COVID-19 and 20,120 healthy images in the validation dataset, and 84 COVID-19 and 1,907 images in the external validation/testing dataset. In training and validation sets, the normal to COVID-19 ratio is 1341:1.

**Fig. 3.**
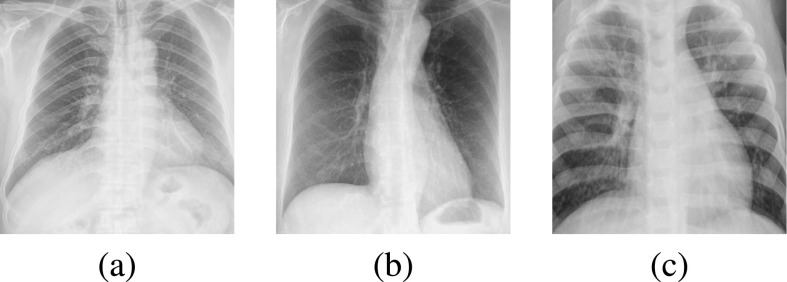
Examples images of (a) healthy patient, (b) COVID-19 patient, and (c) bacterial pneumonia patient.

We also obtained pneumonia images to assess the model performance in distinguishing COVID-19 from healthy or pneumonia (bacterial and viral) images. From the ChestX-Ray8 dataset, we added 944 pneumonia images to the training set and 472 images to the validation set. From the Kermany *et al.* dataset [48], we added 4,273 images to the testing set. Example images are presented in Fig. 3.

#### 3) CT Datasets

**Fig. 4.**
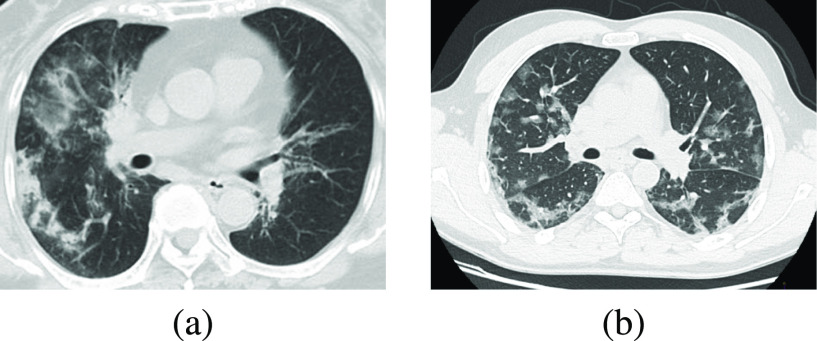
Examples of (a) non-COVID-19 and (b) COVID-19 positive CT scans.

A private CT dataset comprising subjects with or without COVID-19 was collected and used with all relevant approval. This dataset consists of 1919 non-COVID-19 images and 30 COVID-19 images. We divided this the dataset into 909 non-COVID-19 and 15 COVID-19 for training, 303 non-COVID-19 and 5 COVID-19 for validation, and 707 non-COVID-19 and 10 COVID-19 for testing. The ratio of non-COVID-19 to COVID-19 images is at 60.6:1. Example images are shown in Fig. 4. We then externally validated the trained model on data from two publicly available datasets, using the same exclusion criteria as for X-ray. The first dataset is taken from the COVID-CT-Dataset [51], the second from the Italian Society of Radiology [49]. From the COVID-CT-Dataset, we extracted 169 non-COVID images and 100 COVID-19 images. From the Italian Society of Radiology dataset, we extracted 99 COVID-19 images.

## IV. Experimental Setup

### A. Computing

All experiments were performed on a Linux machine running Ubuntu 18.04, with 32 GB of memory and a 12 GB Titan X graphics card. Models were developed using Keras 2.3.1 [52] with Tensorflow 2.1.0 [53] as the backend. Analysis of the results was carried out in R [54] using the pROC [55], reportROC [56], and PredictABEL [57] packages. Source code is available at [github link, will be uploaded in time for publication].

The discriminative performance was evaluated using the AUC, sensitivity, and specificity, with the optimum chosen based upon Youden's index. We assessed reclassification using the net reclassification index (NRI). DeLong's method [58] was used to construct 95% confidence intervals for AUC, and 2000 sample bootstrapping for sensitivity and specificity. Difference in AUCs was identified using DeLong's test [58].

Model training was conducted with 250 epochs with model checkpoints and early stopping with a patience of 10, to prevent overfitting. The best model was chosen based on the validation AUC. The initial learning rate was set to 1e-4 and was reduced to two-thirds if the validation AUC did not improve after five epochs. All hyperparameters were kept the same for all experiments.

## V. Results

In this section, we will present our results from the experiments that we have described above. We will compare the proposed GEV activation function with the previously used sigmoid activation. We begin with results in the toy healthy vs. pnemonia dataset, to show how the ratio of imbalance affects the results. Results on X-rays are then given for healthy vs. COVID-19 images, then for healthy and pneumonia vs. COVID-19. We also demonstrate the performance of the activation function on CT images. The proposed activation function can be combined with other long-tailed distribution methods to improve accuracy further; to demonstrate this, we also use class weights in the model training. GradCAMs [33] were used to highlighted areas that the model believes more important for the prediction where more bright yellow means more relevant while blue means less relevant.

### A. Balanced Data

We began by comparing the GEV function with the sigmoid activation in a balanced dataset of X-ray images. We then increase the ratio of imbalance to assess at what point the proposed activation function becomes useful. We also compare with a popular resampling technique, namely oversampling, which repeats cases from the smaller dataset to rebalance the set.

When data is balanced we observe no significant difference in performance between the sigmoid activation and the proposed GEV activation, the same is also true at a small imbalance ratio of 1:10, with oversampling also showing no significant difference. When the imbalance is increased to 1:25, the GEV provides a statistically significant improvement in AUC and sensitivity over the sigmoid activation; however, using oversampling also improves sensitivity. There is a non-significant increase in performance when using GEV rather than oversampling. At a ratio of 1:50, GEV again provides a significant increase in AUC and sensitivity; however, oversampling overfits and classifies almost all images as negative. Here, we see that resampling strategies may lead to overfitting, even at relatively low levels of imbalance.

Results are displayed in Table I, with the learned GEV activation function for the ratio 1:50 displayed in Fig. 2 compared to the sigmoid function and two other possible GEV curves.

**TABLE I:** Results of Pneumonia vs. Healthy With Different Ratios of Imbalance. Sigmoid Indicates no Balancing Method Used, OS Uses Oversampling With the Sigmoid Activation, GEV Indicates the Proposed GEV Activation. Values for OS Sigmoid With Balanced Data Are Omitted as no Resampling Strategy Is Needed Here

Ratio P:N	Method	AUC	Sens	Spec
1:1	Sigmoid	0.993 (0.990, 0.996)	0.962 (0.947, 0.978)	0.962 (0.947, 0.978)
	OS	-	-	-
	GEV	0.994 (0.991, 0.997)	0.966 (0.943, 0.980)	0.967 (0.953, 0.975)
1:10	Sigmoid	0.975 (0.966, 0.983)	0.919 (0.897, 0.941)	0.947 (0.929, 0.965)
	OS	0.981 (0.975, 0.970)	0.930 (0.909, 0.950)	0.933 (0.913, 0.953)
	GEV	0.985 (0.978, 0.991)	0.959 (0.943, 0.975)	0.959 (0.943, 0.975)
1:25	Sigmoid	0.916 (0.899, 0.933)	0.792 (0.760, 0.825)	0.954 (0.937, 0.971)
	OS	0.959 (0.948, 0.970)	0.875 (0.848, 0.902)	0.923 (0.901, 0.944)
	GEV	0.971 (0.961, 0.981)	0.919 (0.897, 0.941)	0.966 (0.951, 0.980)
1:50	Sigmoid	0.609 (0.575, 0.644)	0.419 (0.378, 0.459)	0.931 (0.911, 0.952)
	OS	0.529 (0.520, 0.539)	0.058 (0.039, 0.077)	1.0 (1.0, 1.0)
	GEV	0.941 (0.928, 0.954)	0.828 (0.798, 0.859)	0.937 (0.917, 0.956)

### B. COVID-19 vs. Healthy

**Fig. 5.**
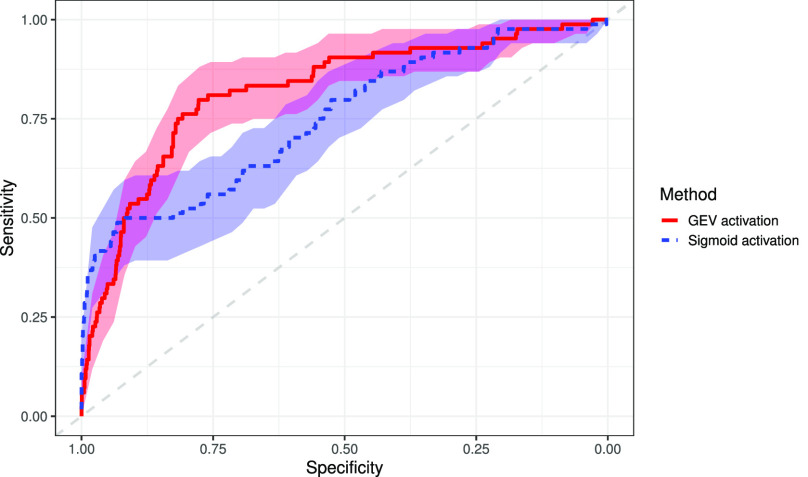
Receiver operating characteristic curve for COVID-19 vs. healthy X-ray images using both the traditional sigmoid activation and the proposed GEV activation, with 95% confidence bands.

**Fig. 6.**
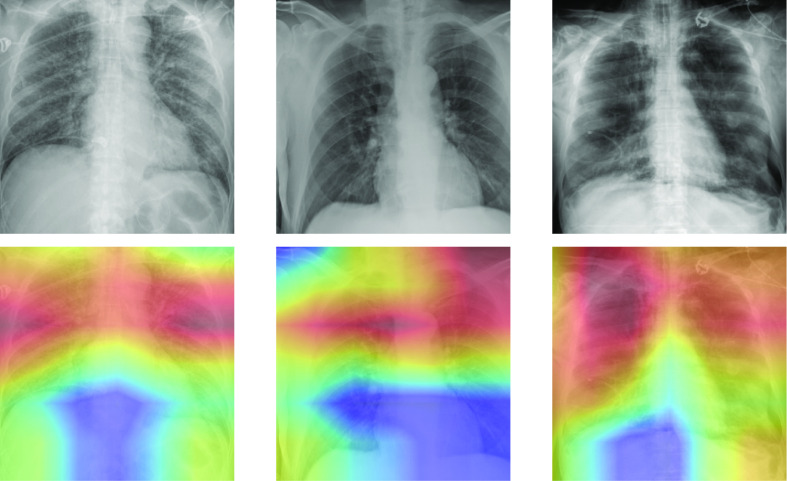
CAMs of some testing images for the classification of COVID-19 vs. healthy images. Red areas show which parts of the image the algorithm believes to be useful in the diagnosis. The algorithm appears to identify the correct regions of interest, concentrating on areas with GGO and consolidation.

In the binary classification between COVID-19 and healthy, the proposed method achieves an AUC of 0.820 (95% confidence interval [CI]: 0.770, 0.870), 0.798 (95% CI: 0.712, 884) sensitivity, and 0.778 (95% CI: 0.759, 0.796) specificity in the testing dataset. The sigmoid activation attained an AUC of 0.750 (95% CI: 0.690, 0.809), sensitivity of 0.488 (95% CI: 0.381, 0.595), and specificity of 0.932 (95% CI: 0.921, 0.944). Results are presented in Table II, with the ROC presented in Fig. 5. DeLong's test for a difference in AUC gave a p-value < 0.05, indicating that our AUC value is significantly higher than that of using sigmoid function at the 95% confidence level. The NRI between the model using the sigmoid activation and the proposed GEV activation is 0.2845 (95% CI: 0.1664, 0.4026), indicating a significant improvement in the classification of GEV activation. Saliency maps were produced to show which areas of the image were considered relevant by the algorithm, shown in Fig. 6.

**TABLE II:** Results of COVID-19 vs. Healthy X-Ray Images. 95% Confidence Intervals Are Given in Brackets

**Activation**	**AUC**	**Sensitivity**	**Specificity**
Sigmoid			
	0.750 (0.690, 0.809)	0.488 (0.381, 0.595)	0.932 (0.921, 0.944)
GEV			
	0.820 (0.770, 0.870)	0.798 (0.712, 0.884)	0.778 (0.759, 0.796)

### C. COVID-19 vs. Healthy and Pneumonia

**Fig. 7.**
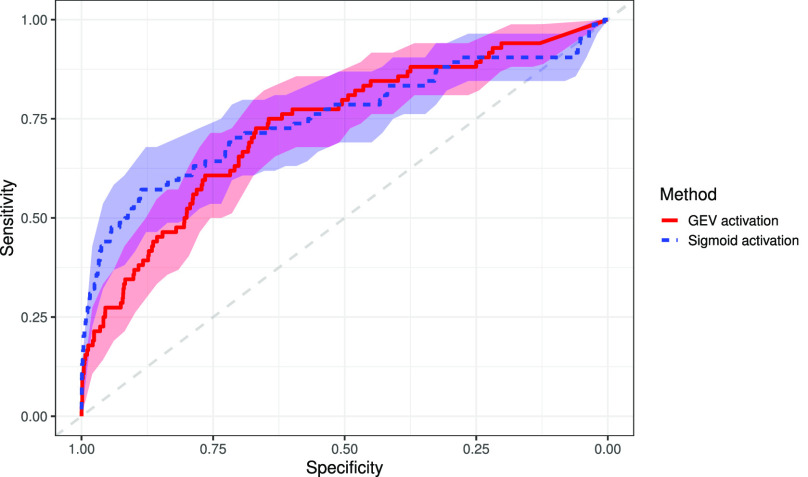
Receiver operating characteristic curve for COVID-19 vs. healthy and pneumonia X-ray images using both the sigmoid activation and the proposed GEV activation, with 95% confidence bands.

**Fig. 8.**
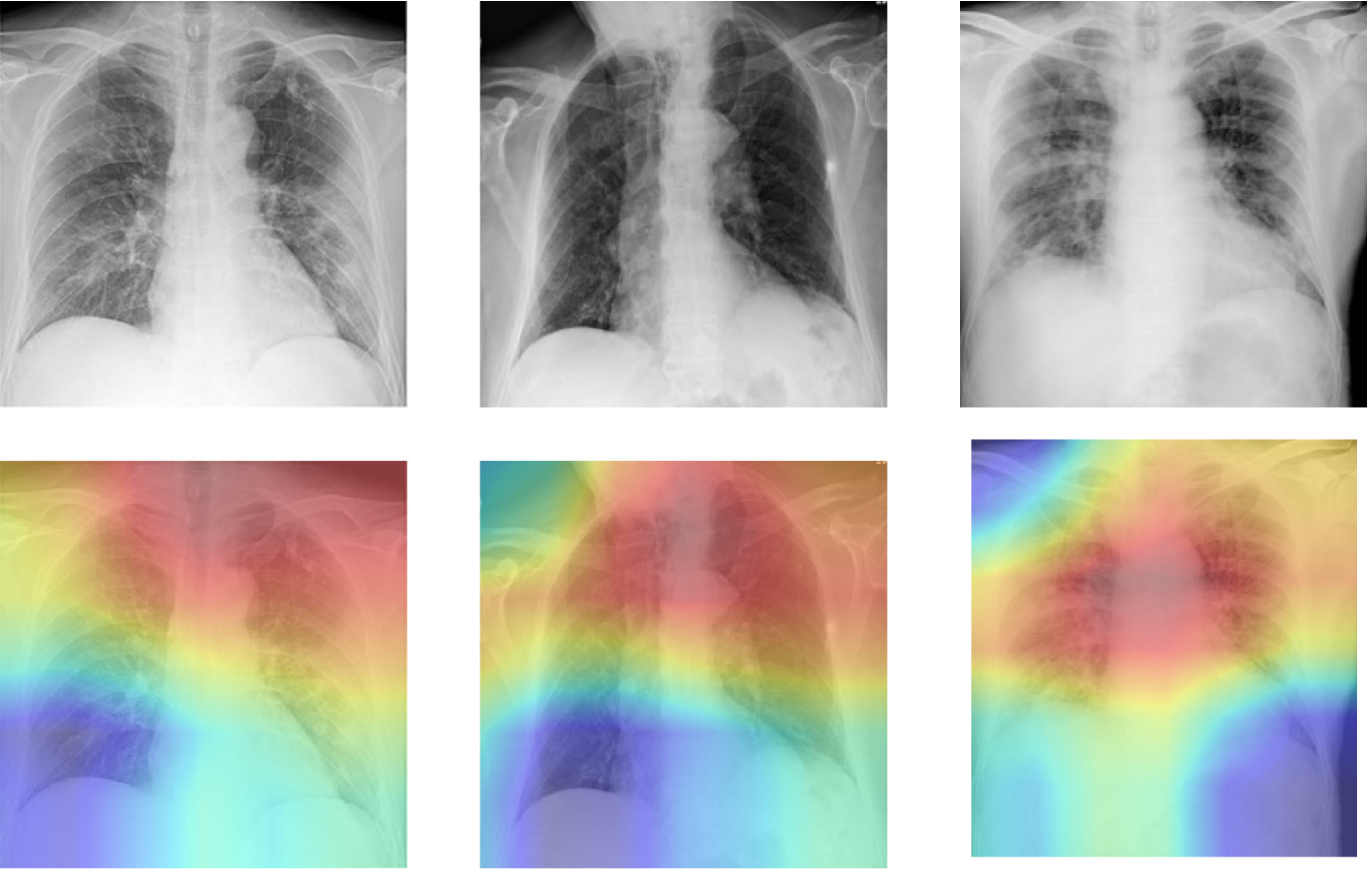
Saliency maps of some testing images for the classification of COVID-19 vs. healthy and pneumonia X-ray images. Red areas show which parts of the image the algorithm believes to be useful in the diagnosis.

For the classification of COVID-19 versus healthy and pneumonia, our method achieves an AUC, sensitivity, and specificity of 0.731 (95% CI: 0.672, 0.790), 0.726 (95% CI: 0.631, 0.822), and 0.669 (95% CI: 0.657, 0.681), respectively. The sigmoid activation attains an AUC, sensitivity, and specificity of 0.752 (95% CI: 0.684, 0.819), 0.571 (95% CI: 0.466, 0.677), and 0.886 (95% CI: 0.878, 0.894), respectively. Delong's test [58] shows a non-significant difference in AUC (p = 0.370); however, the NRI suggests a significant improvement in classification (p < 0.0001), when using the GEV activation. Results are presented in Table III, with the ROC curve shown in Fig. 7. Fig. 8 presents some example saliency maps that highlight which parts the algorithm believes to be useful in the diagnosis.

**TABLE III:** Results of COVID-19 vs. Healthy and Pneumonia X-Ray images. 95% Confidence Intervals Are Given in Brackets

**Activation**	**AUC**	**Sensitivity**	**Specificity**
Sigmoid			
	0.752 (0.684, 0.819)	0.571 (0.466, 0.677)	0.886 (0.878, 0.894)
GEV			
	0.731 (0.672, 0.790)	0.726 (0.631, 0.822)	0.669 (0.657, 0.681)

### D. Classification Using CT

**Fig. 9.**
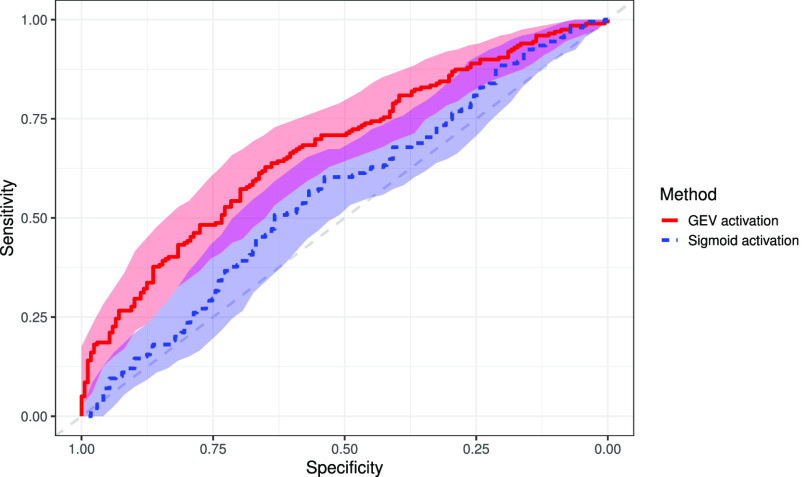
Receiver operating characteristic curve for COVID-19 vs. other CT images using both the sigmoid activation and the proposed GEV activation.

**Fig. 10.**
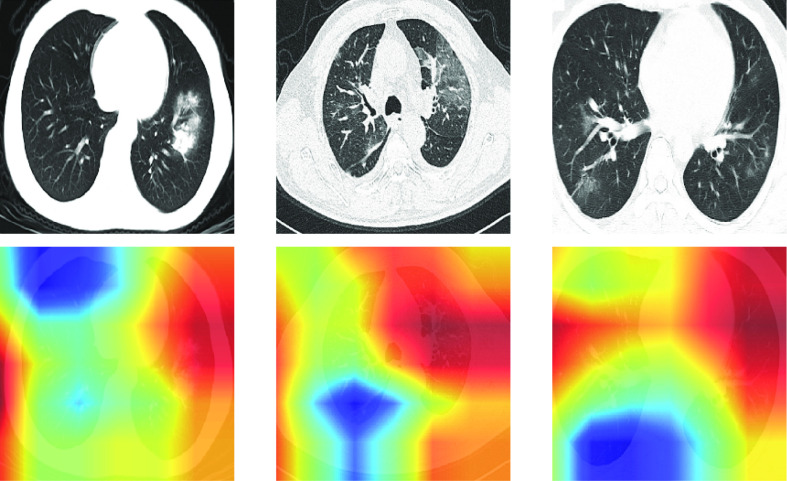
CAMs of some testing images for the classification of COVID-19 vs. other CT images. Red areas show which parts of the image the algorithm believes to be useful in the diagnosis.

To demonstrate the generalizability of our proposed activation function, we also applied the proposed GEV activation function on computerized tomography images. Here, we classify COVID-19 vs. other patients. The GEV activation achieves an AUC, sensitivity, and specificity of 1.0 (95% CI: 1.0, 1.0), 1.0 (95% CI: 1.0, 1.0), and 1.0 (95% CI: 1.0, 1.0), respectively. The sigmoid activation attains an AUC, sensitivity, and specificity of 0.6498 (95% CI: 0.444, 0.855), 0.40 (95% CI: 0.096, 0.704), and 0.908 (95% CI: 0.887, 0.929), respectively. External validation using the datasets previously described attained an AUC, sensitivity, and specificity of 0.675 (95% CI: 0.621, 0.730), 0.628 (95% CI: 0.561, 0.695), and 0.651 (95% CI: 0.579, 0.723), respectively, using the GEV activation. The sigmoid activation attained 0.561 (95% CI: 0.502, 0.620), 0.0 (95% CI: 0.0, 0.0), and 1.0 (95% CI: 1.0, 1.0), for AUC, sensitivity and specificity respectively. The sigmoid activation classified all images as non-COVID giving perfect specificity with 0.0 specificity. DeLong's test indicated a significant difference in AUCs at the 95% confidence level (p = 0.002), the NRI also indicated a significant improvement in classification. Results are shown in Table IV with the ROC curve shown in Fig. 9 and CAMs maps shown in Fig. 10. The CAMs shown here identify correct regions; however in the first and second CAMs, the left side of the image is also identified. The lungs were not segmented, so there is a lot of noise outside of the region of interest. This suggests that segmenting the lung and masking all other regions before classification may be useful.

**TABLE IV:** Results of COVID-19 vs. Healthy in the CT Testing Dataset. The Sigmoid Activation Classified all Images as Non-COVID, Resulting in the Unusual Confidence Intervals Given in Brackets

**Activation**	**AUC**	**Sensitivity**	**Specificity**
Sigmoid			
	0.561 (0.502, 0.620)	0.0 (0.0, 0.0)	1.0 (1.0, 1.0)
GEV			
	0.675 (0.621, 0.730)	0.628 (0.561, 0.695)	0.651 (0.579, 0.723)

### E. Extension to Other Networks

To demonstrate that the proposed network extends to other neural networks, we reperform the COVID-19 versus healthy patients experiments using another pretrained neural network, namely MobileNet. We chose MobileNet as it is specifically designed to be smaller and more deployable than other deep neural networks. As COVID-19 diagnostic models are deployed, more computationally efficient algorithms will be needed to reduce the cost to healthcare services. MobileNet uses depth-wise separable convolutions to reduce the computational complexity of the network, with only a small reduction in accuracy. The network is designed

**Fig. 11.**
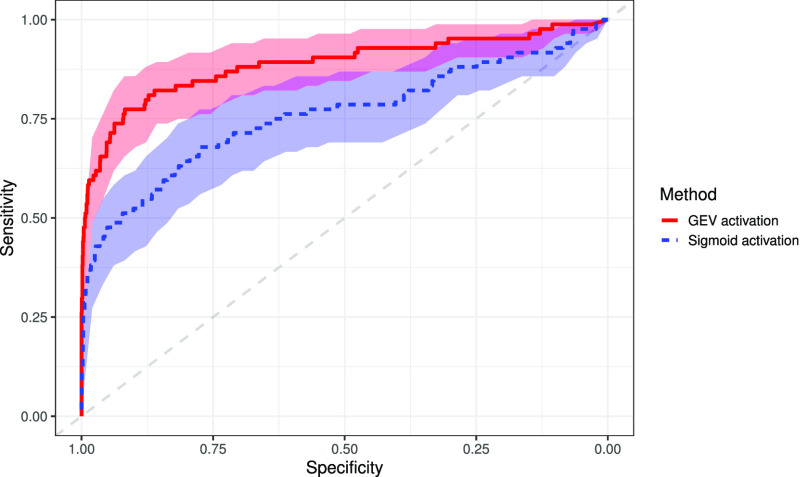
Receiver operating characteristic curve for COVID-19 vs. other CT images using both the sigmoid activation and the proposed GEV activation.

The sigmoid activation attains an AUC, sensitivity, and specificity of 0.759 (95% CI: 0.692, 0.827), 0.679 (95% CI: 0.579, 0.778), and 0.777 (95% CI: 0.758, 0.795), respectively. While the GEV activations attains an AUC, sensitivity, and specificity of 0.888 (95% CI: 0.838, 0.937), 0.774 (95% CI: 0.684, 0.863), and 0.918 (95% CI: 0.905, 0.930), respectively. DeLong's method indicated a non-zero difference in AUC (p < 0.0001) and the NRI shows a significant improvement in classification (p < 0.0001). The ROC curve is shown in Fig. 11.

## VI. Discussion

In this work, we have proposed a novel activation function inspired by extreme value theory and studied its significance for the diagnosis of COVID-19. The proposed GEV activation is better suited to highly unbalanced data than the commonly used sigmoid activation. Experiments conducted using both X-ray and CT imaging to diagnose COVID-19 have shown that the GEV activation function significantly increases sensitivity compared to sigmoid.

On a balanced dataset, we found no significant difference between the GEV activation and the sigmoid activation. As the ratio of imbalance between the classes increased, we found that the GEV activation provided improved sensitivity, accounting for the relatively small number of positive images. Compared to oversampling, when the ratio of imbalance was low, there was no signifcant difference to the GEV activation. However, at a ratio of 1:50, oversampling lead to overfitting, with worse performance that using the sigmoid activation alone. This highlights a common criticism of resampling procedures.

In the diagnosis of COVID-19, hospitals are beginning to use a chest X-ray before CT, as an X-ray is much faster, cheaper, and the machine is much easier to clean than the CT scanner after use. Any negative patients are sent for a CT to confirm the negative diagnosis; hence, it is vital to improve the sensitivity of X-ray algorithms to reduce the number of unnecessary CTs. It is reported that the sensitivity of X-rays for the diagnosis is only about 0.69 in clinical settings [59]. It is expected that automated AI diagnosis algorithms can help to improve sensitivity.

For the COVID-19 vs. healthy experiment, the GEV activation function gave a statistically significant increase in AUC, with a significantly improved sensitivity while maintaining a reasonably high specificity. In the COVID-19 vs. healthy and pneumonia experiment, the GEV activation had a non-significant decrease in AUC with the benefit of increasing sensitivity significantly.

In the CT experiment, the internal testing set was too small to be meaningful (10 patients), and the model using the GEV activation attained perfect performance, while the model using the sigmoid activation was equivalent to random chance. In the external validation set, the GEV model had much lower performance; however, the model still improved significantly over the sigmoid model, which just classified all images as non-COVID.

We have also shown that the proposed activation function generalizes to other networks and improves over the previously used sigmoid activation. In this case, the proposed activation function gave a non-significant increase in sensitivity but gave a significant increase in specificity at the 95% confidence level.

In all experiments, while the AUC was not necessarily improved, the sensitivity was always increased and the NRI indicated that the classification was significantly improved upon when using the GEV activation.

COVID-19 is a new challenge, as yet there is no consensus on performance requirements for any diagnostic models. From our experience, it is believed high sensitivity with a reasonably good specificity would be a preferred option; any false negatives will leave COVID-19 undiagnosed, increasing the risk of spreading this highly infectious disease, while any false negatives may only lead to unnecessary further investigations including CT. Our activation function leads to preferable models over the sigmoid function.

We are cautious to compare out quantitative results with previous work, as summarized in Shi *et al.* [7]. Although the previous models have shown the potential of AI, they often only use a relatively small testing dataset. For instance, the total number of positive cases is between 45 and 100 for the X-ray work [16], [17], [22]. The need for large amounts of training data has led to very few COVID-19 images being used in the testing set. This makes it challenging to determine the model performance and generalizability fully. The proposed GEV activation requires fewer COVID-19 images in the training dataset, meaning we can use more images in the testing set. Although we may not necessarily obtain improved results over previously published COVID-19 diagnostic models, we show that our approach may help to improve those methods, particularly the sensitivity. The other reason for not comparing our model to previous models is that we are not able to obtain the exact same datasets for the experiments, to report the performance. To make a fair comparison, community effort is needed to curate a dataset for reproducible research. It is also of great importance to standardize how results are reported and the measures of a successful model. A systematic review by Wynants *et al.* [8] identified eight studies diagnosing COVID-19 and found that the risk of bias was high in all studies, according to PROBAST [39]. In this work, we have strived to follow best practices for prediction modes, as outlined by PROBAST [39] and TRIPOD [60], and would encourage new prediction models in AI to follow PROBAST and TRIPOD guidelines carefully to increase the robustness of developed models.

Similar to previous studies, the most significant limitation of this study lies in the data used. First, relatively few images are available in the public domain. The data that is available is curated by several initiatives, and typically they are COVID-19 images, without matching negative controls. Currently, non-COVID images must be found elsewhere, and these often use different protocols, making the images slightly different in appearance. Secondly, those that are available are often of low quality, for example: the COVID-19 Image Collection is extracted from published papers and reports [13]. Annotations or captions are on many of the images and it is not always possibly to crop these annotations out. The contrast may have been adjusted during the publication process, and some were downsampled, leading to a significant reduction in details. For the healthy X-ray images used here, although we aimed to crop annotations out of every image, with such a large dataset, we were forced to use an automated cropping technique; this may leave some images still with annotations. It is also possible that some labels in the datasets used were wrong. The samples may also not be representative of the overall population. When we developed our CT model (unreported results), we firstly used publicly available datasets for training; the non-COVID-19 images came from a lung cancer screening set, and the COVID-19 came from [13]. We found that performance was near perfect. After further investigation, we found that the algorithm was learning the appearance of the images and not the features of COVID-19. This highlights the dangers of black-box models and the need for visualization techniques such as saliency maps. We then obtained higher quality images for training, and those results are presented here. Third, many datasets do not contain clinical or demographic information, such as diagnostic test used, age, or gender; these could be used to further imporve models. There is a lack of information on how the image is generated from the raw data. For instance, different window functions will produce different appearance of the same CT. These need to be standardized if there is to be a public dataset being made available in the future.

Although we only considered COVID-19 here, the method can be used whenever one class significantly outweighs the other, such as is the case in rare diseases. Future work is needed, both in COVID-19 and other diseases, to confirm the benefit of the proposed activation function and to assess the situations in which it is preferable over the sigmoid activation. In our experiments, we combined the GEV activation with class weights; Other experiments without class weights experienced a loss of model performance (unreported results). This suggests that a combination of methods may be the best strategy. The effect of other data-efficient methods also needs to be assessed. The proposed method also needs to be extended to multiclass problems. For COVID-19 diagnosis, the proposed activation function needs to be tested on improved base networks to provide improved diagnostic performance.

Although only diagnosis is considered here, CT is likely to play an important role in the treatment and in monitoring the progression of COVID-19. Future work is needed to consider how AI can aid clinicians in decision making for the treatment of COVID-19.

This work, along with previous work, has displayed the potential of AI in the diagnosis of COVID-19, these algorithms will be used as either standalone tools or as diagnostic aids to existing systems, to support decision making. Any methods deployed must first be appropriately validated in clinical settings to obtain regulatory approval

## VII. Conclusion

We propose an activation function based on the generalized extreme value distribution. The GEV activation improves model performance in binary classification when one class significantly outweighs the other. The method is applied to a COVID-19 dataset and improves upon a standard pretrained network using the sigmoid activation. Future models using highly unbalanced data may benefit from using the proposed activation function. We hope these models could support better management for COVID-19 with improved sensitivity.
